# Genetic Variability Related Behavioral Plasticity in Pikeperch (*Sander lucioperca* L.) Fingerlings

**DOI:** 10.3390/ani15152229

**Published:** 2025-07-29

**Authors:** Ildikó Benedek, Béla Urbányi, Balázs Kovács, István Lehoczky, Attila Zsolnai, Tamás Molnár

**Affiliations:** 1 Department of Animal Breeding, Institute of Animal Breeding Sciences, Hungarian University of Agriculture and Life Sciences, H-7400 Kaposvár, Hungary; benedek.ildiko@uni-mate.hu (I.B.);; 2 Albert Kázmér Faculty of Mosonmagyaróvár, University of Győr, Var Square 2., H-9200 Mosonmagyaróvár, Hungary; 3 Department of Molecular Ecology, Institute of Aquaculture and Environmental Safety, Hungarian University of Agriculture and Life Sciences, H-2100 Gödöllő, Hungary; 4 National Centre for Biodiversity and Gene Conservation, Institute for Farm Animal Gene Conservation, H-2100 Gödöllő, Hungary; 5 Department of Applied Fish Biology, Institute of Aquaculture and Environmental Safety, Hungarian University of Agriculture and Life Sciences, H-7400 Kaposvár, Hungary

**Keywords:** pikeperch, pellet consumption, neutral genetic diversity, selection, microsatellite, individual heterozygosity

## Abstract

Background: The relationship between genetic diversity and fitness is well understood, but few studies have investigated how behavior influences genetic diversity, or vice versa. We investigated the relationship between feeding behavior (on a pelleted diet) and genetic diversity in pikeperch, a piscivorous species. Methods: A total of 135 juvenile pikeperch from the same stock were grouped into three behavioral groups: pellet consuming, pellet refusing, and cannibalistic. Eighteen microsatellite markers were used to characterize the genetic diversity and structure of individuals. Results: The juveniles were classified into two genetic clusters: one dominated by pellet-consuming individuals and the other by pellet-refusing individuals containing equal proportions of cannibal individuals. Three of the microsatellite markers were under selection, but only one showed significant genetic segregation between the groups. For this marker, the pellet consumption was associated with low fragment length. Individual multilocus heterozygosity was significantly higher in the pellet-refusing group. Conclusions: These results suggest that pellet consumption acts as an uncontrolled selective force during domestication, influencing the genetic variability of domesticated populations. The ability to habituate to pellets has a significant genetic basis. Cannibalism does not affect genetic variability, and the emergence of the trait is independent of the propensity to consume pellets.

## Simple Summary

In addition to the traits that are directly influenced by humans, many other traits change as a result of the cultural environment during domestication. The ability to adapt to a new environment varies greatly between individuals and depends on genetic diversity. In the intensive rearing of fish, pelleted feed is used. For piscivorous species such as pikeperch, this deviates significantly from their natural diet and influences the survival of their larvae. In our study, we investigated genetic variation among individuals consuming pellets, those refusing to eat pellets, and those adopting an alternative strategy (cannibalism). Our results show that individuals that do not consume pellets have higher genetic variation than those that do, and that the two groups are genetically distinguishable, as confirmed by a marker under positive selection. These results suggest that pellet habituation acts as an uncontrolled selective force during domestication, influencing the genetic variability of domesticated stocks. Cannibal individuals exhibited variability comparable to that observed in the initial population, and they originated from the same proportions of the two genetic clusters that define the other two groups. This indicates that the emergence of the trait is independent of the ability to consume pellets.

## 1. Introduction

Modern aquaculture is a dynamic agricultural sector, dependent on introducing new species to cultivation (domestication) and developing new tools and methods to improve production. During domestication, strong genetic selection pressure to adapt to new environments is exerted in the artificial conditions created by humans, which differ greatly from the natural environment [1]. The species in question must adapt to the cultural environment and the challenges created by artificial selection (‘exploitative aspects’). A functional phenotype, involving both morphology and behavior, evolves and develops as a single entity [2], even though domestication (direct selection) usually targets morphological traits. Consequently, the behavior of the domesticated type changes, and the new traits that evolve are not necessarily adaptive in the natural environment [3].

The association between behavior and other traits is well evidenced and framed by the Pace-of-Life Syndrome (POLS) hypothesis [4]. Sih and Del Giudice [5] extended the POLS hypothesis to include cognition, based on the relationship between fast and slow behaviors and cognitive speed and accuracy. The concept of a ‘cognitive syndrome’ proposes that individuals exhibit diversity in their problem-solving ability, which is related to their behavioral type. Resilient individuals respond quickly to environmental changes and tend to be cautious and reactive, whereas bold, proactive individuals exhibit more routine and predictable behavior over time [6].

The Percidae family is one of the most important for the diversification of freshwater aquaculture. The pikeperch (*Sander lucioperca* L.), a large species within this family, plays a key role in European aquatic ecosystems [7] and provides valuable resources for recirculating aquaculture systems (RAS). Like other percid species, pikeperch follow ontogenetic patterns in their foraging, transitioning from zooplankton to piscivory at sizes of 11.0 mm in the laboratory and 13.5 mm in the wild, which shows high individual variability within the species [8,9]. Weaning onto pelleted diets used in intensive rearing occurs at this stage for both larval and pond-reared fry. As this species is an obligate piscivore, changes in foraging behavior (pellet consumption) can significantly impact its efficiency when hunting live prey [10,11]. Conversely, new traits under intensive conditions may lead to alternative foraging strategies emerging [12]. However, acceptance of a pelleted diet is a learning process, the outcome of which depends heavily on an individual’s life history and cognitive type [13].

During the ontogenetic switch in foraging behavior, significant differences in gene expression were observed between the two groups (plankton consumers and fish consumers), highlighting the importance of the complex genetic background and individual differences involved in this process [14]. The differentially expressed genes were those involved in brain development during the juvenile stage and those influencing appetite. As individuals from both groups were found simultaneously, it can be assumed that differences in foraging are due not only to environmental influences but also to genetic variability.

Advances in molecular genetics have also made it possible to explore the links between the diversity observed in traits and genetic diversity. The estimation of the relationship between individual genetic variation and individual measures of different traits (affecting adaptability) is known as heterozygosity-fitness correlations (HFC) [15]. HFCs affect various aspects of fitness, including the number and survival of offspring, resistance to parasites, and reproductive success, as well as behavior [16]. Population genetics studies on pikeperch indicate that the genetic variability of intensively reared stocks (fed on pellets) varies greatly depending on the origin of the initial population [17,18] and the presence of an appropriate management or breeding program to mitigate the effects of genetic drift, which can significantly reduce genetic diversity [19,20]. However, the extent to which uncontrolled selection effects during domestication influence genetic diversity, and how genetic diversity affects these processes, is not well understood. One of the strongest uncontrolled selection effects during intensive rearing is the weaning of larvae onto pellets.

In our study, we investigated genetic diversity among individuals with different foraging abilities during the pellet-weaning period. Our primary objective was to determine how population genetic diversity indices evolve in groups formed on the basis of pellet-feeding ability (pellet-feeding, pellet-non-feeding, and cannibalistic) and to observe differences in individual heterozygosity values that might confirm the existence of a heterozygosity-fitness correlation (HFC). Furthermore, we aimed to investigate whether genetic structure could be observed according to foraging ability or strategies and whether genetic selection for the markers used could confirm this structure.

## 2. Materials and Methods

### 2.1. Ethical Approval

The protocol was approved by the Committee on the Ethics of Animal Experiments of the Hungarian University of Agriculture and Life Sciences Kaposvár Campus (permit number: 3/2016-MÁB).

### 2.2. Experimental Animals

A total of 135 pikeperch fingerlings with an average body weight of 14.76 ± 5.60 g were involved in the experiment. These animals had previously been used in a study on feeding behavior, in which the rearing and feeding training conditions were described in detail [13]. Briefly, during weaning, the pond-reared individuals were conditioned to consume pellets by gradually reducing the amount of live feed provided alongside additional pellets, as described by [21,22]. The weaning to pelleted feed was carried out in 300-liter tanks containing 2000 pond-reared fry at a stocking density of 0.8 fry per liter. The weaned fish were then reared in 65-liter aquaria (60 × 30 × 30 cm) working within an RAS. The temperature was 21 ± 0.5 °C, the photoperiod was set to 12/12 h, and the dissolved oxygen was 7.8 ± 0.4 mg/L. After weaning, the 135 individuals were divided into three groups: pellet feeders (group PF, N = 65), non-feeders (group PNF, N = 57), and cannibals (group C, N = 12). Individuals were classified into three groups using the following method. During the weaning period, feeding took place at the same time every day: a mixture of pellets and chopped Tubifex was provided at 8 am and 6 pm. On the seventh day of each week, the fish were only fed pellets in the morning. Those that accepted the pellets were satiated, recognizable by their large, yellowish bellies. These fish were used to create the PF group. At the end of the three-week process, those that refused the pellets were formed into the PNF group. Throughout the study, individuals that preyed on their companions were separated. These individuals were easily identifiable before feeding in the morning, as eating a fish of the same size resulted in a large belly, with the tail of the prey often hanging out of the mouth.

### 2.3. DNA Extraction and Microsatellite Analysis

During sampling, experimental fish were handled using size-appropriate nets and anesthetized with clove oil (10 drops/10 L of water) to reduce stress. Following sampling, fish were placed in aerated fresh water. The fin clip samples were collected in 96% ethanol and stored at −80 °C until processing. Genomic DNA was extracted using DNeasy Blood and Tissue kits (Qiagen, Hilden, Germany), following the manufacturer’s protocol. The quantity and quality of the DNA were checked using a Maestro NanoDrop MN-913 spectrophotometer (MaestroGen, Hsinchu City, Taiwan). The DNA concentration of each sample was adjusted to 50 µg/mL.

A total of 18 microsatellite DNA markers were used: MSL1, MSL2, MSL3, MSL5, MSL6, and MSL9 [23]; Svi-4, Svi-6, Svi-L7, Svi-L8, and Svi-18 [24]; Pfla-L3, Pfla-L8, and Pfla-L9 [25]; and Za038, Za138, Za144, and Za199 [26]. These markers were used to genotype all individuals and were amplified in four multiplex PCRs using NED-, PET-, VIC-, and FAM-end-labelled primers (Table 1).

Amplifications were performed in a reaction volume of 20 µL with AmpliTaq Gold^®^ DNA polymerase (Promega Corporation, Madison, Wisconsin, USA) in Buffer II (100 mM Tris-HCl, pH 8.3; 500 mM KCl). The final reaction conditions for multiplexes A, B, C, and D were as follows: 1× PCR buffer, 1.5 mM MgCl_2_, 200 µM of each dNTP, 1.2 units of Taq DNA polymerase, and 50 ng of genomic DNA template. The concentrations of the forward and reverse primers were as follows: Multiplex A: 0.1 µM MSL1, 0.066 µM MSL3, 0.266 µM MSL5, 0.2 µM MSL6, and 0.2 µM MSL; Multiplex B: 0.2 µM MSL2, 0.1 µM Svi-4, 0.1 µM Svi-62, µM Svi-L7, 0.2 µM Svi-18, and 0.2 µM Pfla-L8; Multiplex C: 0.1 µM Pfla3, 0.25 µM Za138, 0.05 µM Za199, and 0.3 µM Svi-L8; Multiplex D: 0.3 µM Pfla9, 0.1 µM Za038, and 0.2 µM Za144.

The temperature profile for amplifications of multiplexes A, B, and D was as follows: Initial denaturation at 94 °C for 10 min, followed by 35 cycles of 60 s at 95 °C, 90 s at the annealing temperature (56 °C for multiplex A, 55 °C for multiplex B, and 53 °C for multiplex D), and 60 s at 72 °C, then 72 °C for 20 min. The temperature profile for the amplification of multiplex C was as follows: Initial denaturation at 94 °C for 10 min, followed by 5 cycles of 60 s at 95 °C, 90 s at 50 °C, and 60 s at 72 °C; then 30 cycles of 60 s at 95 °C, 90 s at 47 °C, and 60 s at 72 °C; finally, 20 min at 72 °C. The PCR products were stored at 4 °C. PCR was performed in a Px2 PCR apparatus (Thermo Fisher Scientific, Waltham, MA, USA). Products from the multiplex PCR reactions were pooled and analyzed using a GeneScan 600 LIZ internal size standard (Applied Biosystems, Foster City, CA, USA) on a 3500 Genetic Analyzer (Applied Biosystems, Foster City, CA, USA). After the runs, PCR fragment sizes were determined using GeneMapper version 4.0 software (Applied Biosystems, Foster City, CA, USA).

### 2.4. Statistical Analysis

MICRO-CHECKER version 2.2.3 [27] and Monte Carlo simulation (bootstrap) were used to detect and correct the presence of null alleles at each locus. A total of 1000 randomizations were carried out, and the analysis used a 95% confidence interval. Allele number (N_a_), observed and corrected expected heterozygosity (H_o_ and uH_e_), and fixation index (F_IS_) were calculated using GenAlEx 6.5 software [28]. The estimation of allelic richness (AR) and individual allelic richness (ARP) was carried out using the rarefaction procedure implemented in HP-RARE 1.0 software [29]. H_o_ and F_IS_ values were standardized to population sizes using weighted averages for comparisons between populations. Comparisons of indicators of genetic variability were performed using a Mann–Whitney U-test with Bonferroni correction. The significance level was set at 0.016 [30]. Individual levels of multilocus heterozygosity (scoring individuals as heterozygous—1 or homozygous—0 and the average was taken across all loci [31]) were also compared between the three groups using the Kruskal–Wallis test.

We performed a molecular analysis of variance (AMOVA) and calculated pairwise F_ST_ values and their significance using GenAlEx 6.5 software (9999 permutations were used for statistical significance testing). We used the Bayesian algorithm built into the STRUCTURE software package [32,33] to determine population structure. To estimate the most probable number of clusters (K), we used STRUCTURE Selector [34] to determine the highest posterior probability (lnP(D)) and ΔK values (a quantity based on the rate of second-order change of the likelihood function with respect to K), as described by [35]. We set the burn-in to 10^4^ and the number of additional MCMC runs to 10^5^. These calculations were repeated ten times for each K value. The Neighbour-Joining tree was constructed from genotypic distances calculated using GenAlEx 6.5 software and MEGA11 software [36]. The ADegenet 2.1.1.7 package [37] in the R environment (version 4.2.1) was used to perform discriminant analysis of principal components (DAPC) on microsatellite data. To detect the signal of selection, we used three methods: Arlequin 3.5.2 [38], BayeScan v2.01 [39], and lnRH [40].

## 3. Results

### 3.1. Genetic Diversity

The diversity data for the original stock and the three subgroups are presented in Table 2. Neither the observed nor the expected heterozygosity differed among the groups. The inbreeding coefficient (F_IS_) was low and negative in both the original population and the subpopulations and did not differ between groups. The mean allele number was significantly higher in the non-pellet-consuming group than in the cannibal group (probably due to the smaller number of individuals in the latter group), while the pellet-consuming group was intermediate. This difference was not observed for the effective allele number or allelic richness, and the values were not significantly different. In contrast, private allelic richness showed statistically significant differences, with the group not consuming pellets having the highest allelic richness and the other two groups having significantly lower values.

### 3.2. Genetic Structure

The AMOVA results indicated a variance of 7.37% between the groups. Pairwise F_ST_ values were significant (*p* < 0.05) in all cases. The highest value (0.098) was observed between the PNF and PF groups, while lower values were found between the PNF and C groups (0.038) and the PF and C groups (0.024). PF and PNF groups showed low-to-moderate mean differentiation.

**Figure 1:**
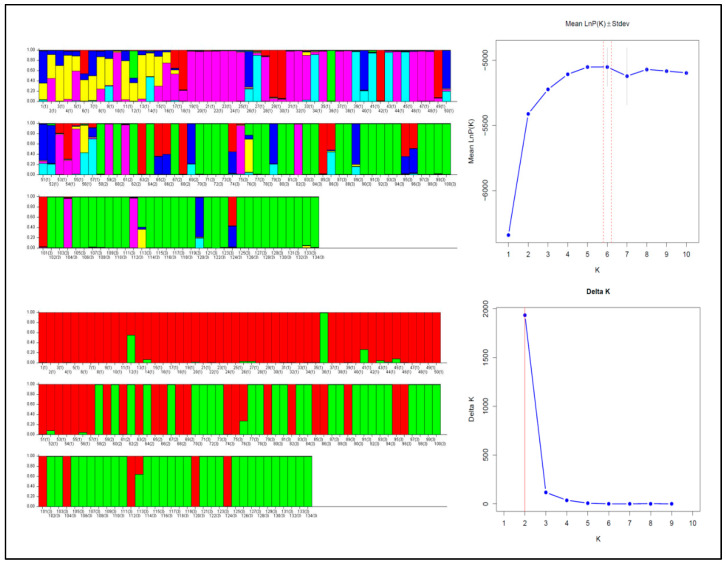
Results of the Bayesian STRUCTURE clustering. The distribution of three (LnPk method) and two (ΔK method) genetic clusters in the individuals of the studied stocks determined by STRUCTURE analysis. The numbered columns indicate individuals, and the numbers in brackets indicate the group: 1-PNF, 2-C, and 3-PF. The different colors in the columns represent different genetic clusters.

This was also confirmed by STRUCTURE analysis. Based on the LnPK method, K = 6 was the most probable cluster number; however, based on the ΔK method used by [35] Evanno et al. (2005), K = 2 was the most probable number when all markers were used in the calculation (Figure 1). In both cases, the PF group largely forms a homogeneous cluster.

**Figure 2:**
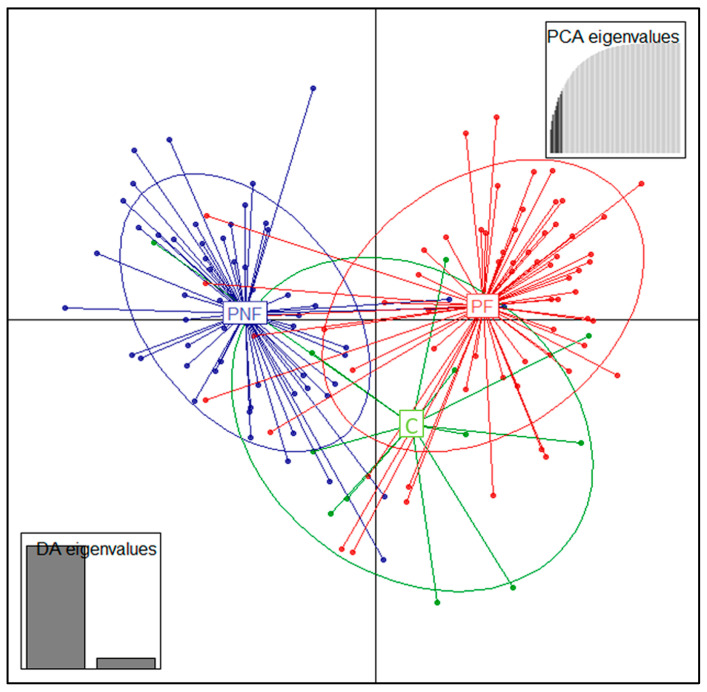
Separation of experimental groups based on DAPC analysis.

The DAPC analysis separated the PF and PNF groups in line with the STRUCTURE results. However, the cannibal group showed a partial overlap with the other two groups (Figure 2).

**Figure 3:**
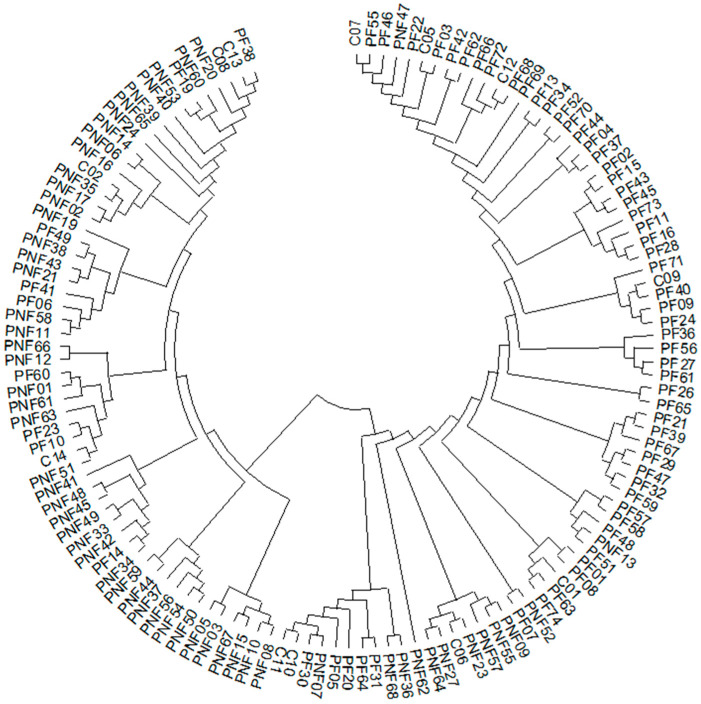
NJ phylogenetic tree of 134 individuals based on genotype distances.

Based on the Neighbour Joining tree (Figure 3), the entire population can be classified into approximately 26 families. The PF and PNF groups are distinct from each other, while the cannibals are scattered amongst them.

### 3.3. Genetic Selection

Three of the 18 loci were identified as outliers by at least one of the methods. The different methods identified 3, 1, and 0 markers using Arlequin, BayeScan, and lnRH, respectively. However, only one outlier (MSL-6) was identified by at least two of the methods (Table 3).

**Figure 4:**
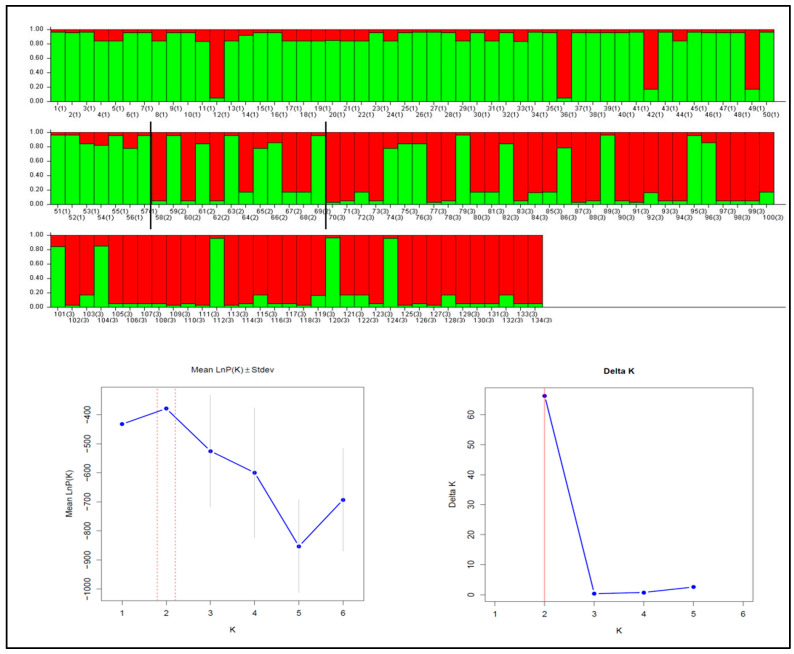
The results of the STRUCTURE analysis using the MSL-1 marker. The numbered columns indicate individuals, and the numbers in brackets indicate the group: 1-PNF, 2-C, and 3-PF. The different colors in the columns represent different genetic clusters.

For the MSL-6 and Svi-6 markers, the detected selection did not result in a significant F_ST_ value between groups. However, significant genetic differentiation between groups was observed for the MSL-1 marker, in addition to a high heterozygosity value. Therefore, we examined the genetic structure of individuals using only this marker (Figure 4). STRUCTURE analysis classified individuals into two clusters using both the LnPK and ΔK methods. These clusters were separated based on the length of the microsatellite marker fragments: the first cluster (green) contained individuals with long fragments (a minimum of 144 base pairs in length), while the second cluster (red) contained individuals with short fragments (up to 140 base pairs). The first cluster predominantly contained members of the PNF group, while the second cluster predominantly contained members of the PF group. Four individuals in the PNF group were classified in the second cluster, while 14 individuals in the PF group were classified in the first. The cannibal group contained an equal number of individuals from both clusters.

When run with the MSL-1 marker, the AMOVA analysis revealed significant between-group variance (F_ST_ = 0.177, *p* < 0.001). Significant group-pairwise F_ST_ values (*p* < 0.001) suggest a separation of the PNF group from both the C group (F_ST_ = 0.132) and the PF group (F_ST_ = 0.213), while the value for the PF and C groups is low (F_ST_ = 0.019).

### 3.4. Individual Heterozygosity Calculated with Neutral Markers and Behavioural Plasticity

Individual multilocus heterozygosity values, calculated using neutral markers, revealed differences based on feeding behavior (Kruskal–Wallis H = 6.071, df = 2, *p* = 0.048; see Table 4). The PNF group exhibited the highest individual heterozygosity among the three behavioral groups, with an average of 10.07 out of 15 loci found to be heterozygous. This value was significantly higher than that of the PF group (9.46) after Bonferroni correction (*p* = 0.041). The value for the cannibal (C) group was intermediate between the other two groups and not significantly different from them. It was also in line with the value measured for the whole baseline population.

## 4. Discussion

Genetic structure analysis using STRUCTURE, DAPC, and AMOVA shows genetic segregation of the PNF and PF groups, with an F_ST_ value of 0.098. This degree of separation is surprising given that behavioral selection was performed on a population considered homogeneous, which had been maintained in isolation for a long time, albeit with a family structure. It is well known that domestication can change genetic variation even within a single generation [41]. In the case of rainbow trout, individuals with the highest fitness in captivity produced offspring with the poorest fitness in the wild, demonstrating the selection of traits that are advantageous in captivity but severely maladaptive in the wild. A genetic study of the species based on these findings showed the differential expression of 723 genes within a single generation, primarily affecting immunity and metabolism during adaptation to hatchery conditions [42].

Although the difference was significant only for allele number and individual allelic richness, the genetic diversity of the pellet-feeding group was found to be lower than that of the pellet-non-feeding group. This suggests that weaning onto a pelleted diet can significantly reduce original genetic diversity. Tsaparis et al. [17] compared wild and domesticated populations using the same microsatellite markers as us. On average, the domesticated populations showed a slightly higher number of alleles (4.75 vs. 4.58) and lower allele richness (3.63 vs. 3.78) than wild populations. However, the genetic diversity of the domesticated populations varied considerably (allelic richness (AR): 2.4–5.5; observed heterozygosity (H_o_): 0.40–0.81; expected heterozygosity (uH_e_): 0.43–0.73), with higher values resulting from the artificial mixing of previously isolated populations. In our study, initial stock diversity for allelic richness (AR = 6.33) exceeded that of European domesticated stocks, while heterozygosity (H_o_ = 0.67; uH_e_ = 0.63) was medium. The values measured for the PF stock can be considered average for all three indicators, corresponding to the values of previously unmixed domesticated stocks. However, it is important to note that diversity indices are also strongly dependent on the number of founder individuals in a population, as demonstrated in our previous study [19].

The relationship between individual genetic diversity and finesse-related traits has long been recognized. Three primary hypotheses have been formulated: the direct effect hypothesis, whereby heterozygote advantage is the result of overdominance at a locus with a direct effect on a given phenotype, and two alternative hypotheses [15]. The first of these is the local effect hypothesis, which is similar to the direct effect hypothesis, except that in this case, the marker is only closely linked to the trait. The second alternative hypothesis is the general effect hypothesis, in which individual heterozygosity represents genome-wide heterozygosity and is related to the degree of individual inbreeding [43]. Functional loci under selection reflect the direct and local effects, respectively, while neutral loci are used to estimate the general effect. In our case, three of the 18 markers used were found to be non-neutral. For two of these markers, only high heterozygosity was observed, and the F_ST_ value remained low, so they were presumably affected by balancing selection. However, for the third marker, directional selection may be at play, whereby a high number of microsatellite repeats results in the behavior not emerging (i.e., an inability to learn to consume pellets). Similarly, an example of a microsatellite marker affecting behavior has been observed in species of the genus Microtus, where the arginine vasopressin 1a receptor (AVPR1A) gene influences the evolution of social behavior [44,45]. Based on STRUCTURE clustering, the four ‘genetically pellet-consuming’ individuals likely failed to adapt due to negative effects during learning. In the PF group, ten of the 14 individuals in the other clusters contained the long allele in heterozygous form. Our experiment showed that the genetic distance between the groups was significantly higher (F_ST_ = 0.213) based on AMOVA using only the MSL-1 marker. This suggests that the marker may be suitable for developing molecular selection for this trait.

The calculated individual multilocus heterozygosity value using neutral markers was significantly lower in the PF group than in the PNF group. Since this indicator estimates the level of genome-wide variation, the general effect theory suggests that the ability to consume pelleted feed (‘novel’ and highly different from previously used feed) is correlated with a higher level of inbreeding. The number of publications investigating the correlation between heterozygosity and behavior (affecting fitness) is low. Based on these studies, we know that heterozygous individuals exhibit behaviors that are advantageous in intra-species competition. These behaviors include being more competitive [31], more aggressive [46,47,48], more dominant [49], and having higher activity levels, as well as being better predators [50]. The behavioral phenotypes listed are typically observed in domesticated individuals [3]. In our case, the ability to learn to consume pellets was associated with lower heterozygosity. However, this finding does not contradict previous studies, as our earlier study [13] found that this learning ability in pikeperch was associated with shy, less active, non-exploratory individuals. Thus, exploratory, bold, and more active individuals in the PNF group had higher heterozygosity, as previously described in other species.

The genetic basis of behavioral change has only been mapped in a few cases. Regarding the model species, the zebrafish (*Danio rerio*), Wright et al. [51] conducted a QTL analysis, comparing domesticated and wild lines. They identified QTLs on chromosomes 9 and 16 associated with boldness and on chromosome 21 associated with antipredator behavior. Similar QTLs have been identified in nine-spined sticklebacks (*Pungitius pungitius*) in relation to exploration, risk-taking, and feeding activity [52]. The effect of domestication on pelleted feeding habituation was investigated in mandarin fish (*Siniperca chuatsi*), with 149 genes being described; most of these were related to memory, vision, and olfactory function [53]. Zarski et al. [54] compared the transcriptomes of pikeperch eggs from wild and domesticated populations, finding that the genes were mainly related to neurodevelopment. They also demonstrated that maternal mRNA shapes embryonic neurodevelopment and presumably behavior [55].

## 5. Conclusions

Our results show that individuals who avoid pellets have greater genetic variation and are genetically distinguishable from those who consume them, as confirmed by a genetic marker under positive selection. This demonstrates that the ability to be weaned onto pellets is significantly influenced by genetic background. Our findings suggest that the habituation to pellets acts as an uncontrolled selective force during domestication, affecting the genetic variability of domesticated stocks. Cannibal individuals exhibited variability similar to that observed in the initial population and originated from the same proportion of the two genetic clusters representing the other two groups. This suggests that the emergence of the trait is independent of a tendency to consume pellets.

## Figures and Tables

**Table 1:** Primers applied in the multiplex reactions (A, B, C and D) in order to genotype the listed loci in the pikeperch groups.

Locus	GenBank ID	Multiplex PCR	Primer Label and Sequence
MSL-1	EF694018	A	F-NED-TGTTTGTCAGCGTCAAGAGG
			R-TTCCGCTCCAACATATCACA
MSL-3	EF694020	A	F-NED-CCGGCATCCATACACCTTAC
			R-CACACCTGTGTCTGCCTAACA
MSL-5	EF694022	A	F-PET-CAATCGCTCTGAGGATGTCA
			R-AAGGGTGGGGAAATTATTCG
MSL-6	EF694023	A	F-FAM-GTCGTCATCGTCAGCACAGT
			R-ACTACACGGGACGCTGGA
MSL-9	EF694026	A	F-VIC-GCATCACTTGCGTCACTTTC
			R-GCAGTCAGTGCTTGAAGTGG
MSL-2	EF694019	B	F-PET-TTTTCACACCGTGCATGACT
			R-ACCCTCAGCCTCTGTGTACG
Pfla L8	AF211833	B	F-FAM-GCCTTATTGTGTGACTTATCG
			R-GGATCTTTCACTTTTTCTTTCAG
Svi18	G36964	B	F-PET-GATCTGTAAACTCCAGCGTG
			R-CTTAAGCTGCTCAGCATCCAGG
Svi4	G36961	B	F-PET-ACAAATGCGGGCTGCTGTTC
			R-GATCGCGGCACAGATGTATTG
Svi6	G36962	B	F-NED-CATATTATGTAGAGTGCAGACCC
			R-TGAGCTTCACCTCATATTCC
Svi-L7	AF144740	B	F-NED-GATGTGCATACATTTACTCC
			R-GCTTTAATCTGCTGAGAAC
PflaL3	AF211828	C	F-FAM-GCCGAATGTGATTGAATG
			R-CGCTAAAGCCAACTTAATG
Za138	HM622317	C	F-VIC-TTCTTTATACAAGAGGAATAGTTGCAG
			R-TTTTTGTGATTGTGCTATTTTAAAGG
Za199	HM622334	C	F-NED-CCTTCCCCTCAAAAGCATGT
			R-AGGAAATGGAAAGGGAATGC
SviL8	AF144741	C	F-PET-GCTTATACGTCGTTCTTATG
			R-ATGGAGAAGCAAGTTGAG
Pfla-L9	AF211834	D	F-PET-GTTAGTGTGAAAGAAGCATCTGC
			R-TGGGAAATGTGGTCAGCGGC
Za038	HM622298	D	F-FAM-TGAATCGCTGCTCTTTCTCA
			R-TATGCAATTACATCGGAGCG
Za144	HM622319	D	F-VIC-GCCCACAATAGCACCGTAAT
			R-TTTGTGAATGTGAGTGAGAGTCAG

**Table 2:** Genetic diversity data for the three subpopulations and the whole original population (mean ± SD).

	N	uH_e_	H_o_	F_IS_	N_A_	Ne	AR	AR_P_
PNF	57	0.63 ± 0.12	0.68 ± 0.16	−0.085 ± 0.13	5.61 ± 1.41 ^a^	3.00 ± 1.00	4.40 ± 0.85	0.56 ± 0.54 ^a^
C	12	0.67 ± 0.12	0.67 ± 0.19	−0.050 ± 0.21	4.44 ± 1.09 ^b^	3.10 ± 1.04	4.44 ± 1.09	0.12 ± 0.18 ^b^
PF	65	0.59 ± 0.17	0.66 ± 0.26	−0.079 ± 0.23	5.50 ± 1.50 ^ab^	2.78 ± 0.87	4.10 ± 0.92	0.23 ± 0.30 ^b^
O	134	0.63 ± 0.14	0.67 ± 0.20	−0.071 ± 0.19	5.18 ± 1.42	2.96 ± 0.96	6.33 ± 1.57	-

PNF: Pellet non-feeding; C: Cannibal; PF: Pellet feeding; O: Original whole stock; N: Number of individuals tested; uH_e_: Corrected expected heterozygosity; H_o_: Observed heterozygosity; F_IS_: Inbreeding coefficient; N_A_: Mean allele number; Ne: Effective allele number; AR: Allele richness; AR_P_: Private allele richness. Values with different letters are significantly different (*p* < 0.05).

**Table 3:** Loci under selection identified by Arlequin, BayeScan, and lnRH.

Locus	Arlequin			BayeScan			LnRH		
	Het	F_ST_	*p*	Prob	Log_10_(PO)	q-Value	PNF/PF	PNF/C	PF/C
**MSL-1**	**0.81**	**0.176**	**0.044**	0.06	−1.23	0.85	−0.82	−0.29	1.11
MSL-3	0.34	0.107	0.231	0.08	−1.08	0.71	0.61	−1.15	0.54
MSL-5	0.81	0.031	0.113	0.06	−1.17	0.81	−0.18	−0.90	1.08
**MSL-6**	**0.74**	**0.009**	**0.036**	**0.88**	**0.85**	**0.12**	0.15	0.92	−1.07
MSL-9	0.73	0.059	0.430	0.05	−1.32	0.88	0.30	0.81	−1.12
Msl-2	0.63	0.205	0.057	0.05	−1.29	0.87	−0.24	−0.86	1.10
Pfla-L8	0.73	0.040	0.253	0.06	−1.21	0.82	0.42	0.73	−1.14
Svi-18	0.63	0.060	0.499	0.04	−1.38	0.89	1.00	−0.01	−1.00
Svi-4	0.78	0.050	0.308	0.07	−1.11	0.75	−0.43	−0.71	1.14
**Svi-6**	**0.71**	**0.010**	**0.046**	0.08	−1.08	0.64	0.72	0.42	−1.14
Svil-L7	0.37	0.052	0.489	0.13	−0.84	0.50	1.14	−0.75	−0.39
PflaL3	0.57	0.033	0.272	0.07	−1.12	0.78	−0.52	−0.63	1.15
SviL8	0.78	0.153	0.088	0.05	−1.26	0.86	0.21	−1.09	0.88
Za138	0.67	0.049	0.377	0.05	−1.32	0.88	−0.38	−0.76	1.13
Za199	0.62	0.118	0.192	0.06	−1.24	0.86	−0.91	−0.15	1.07
PflaL9	0.67	0.085	0.346	0.06	−1.22	0.84	−0.43	−0.71	1.14
Za038	0.68	0.088	0.335	0.04	−1.36	0.89	0.49	−1.15	0.66
Za144	0.85	0.090	0.356	0.04	−1.33	0.89	0.55	−1.15	0.61

Het: Expected heterozygosity; F_ST_: F_ST_ value; Prob: Probability; BayeScan: Prob: Posterior probability of the model with selection; Log_10_(PO): Logarithm of the Posterior Odds; q-value: q-value of the model with selection; LnRH: PNF: Pellet non-feeding, PF: Pellet feeding, C: Cannibal; loci highlighted in bold were considered outliers.

**Table 4:** Individual multilocus heterozygosity values in the behavioral groups, calculated using neutral markers.

Group	N	Mean	SD	Confidence Interval (95%)	
				Lower Bound	Upper Bound
PNF	57	10.07	1.22	9.74	10.39
C	12	9.75	1.21	8.97	10.52
PF	65	9.46	1.32	9.13	9.78
O	134	9.74	1.29	9.52	9.96

N: Number of individuals, PNF: Pellet non-feeding, C: Cannibal, PF: Pellet feeding, O: Original whole stock.

## Data Availability

The raw data of the experiment is available as (Supplementary Material Table S1).

## Supplementary Materials

The following supporting information can be downloaded at: https://www.mdpi.com/article/10.3390/ani15152229/s1. Table S1: The raw microsatellite data of the experiment.

## Abbreviations

The following abbreviations are used in this manuscript:

POLS	Pace-of-Life Syndrome
RAS	Recirculating Aquaculture Systems
HFC	Heterozygosity-Fitness Correlations
PF	Pellet Feeder
NPF	Pellet Non-Feeder
